# oA novel nonparametric approach for estimating cut-offs in continuous risk indicators with application to diabetes epidemiology

**DOI:** 10.1186/1471-2288-9-63

**Published:** 2009-09-10

**Authors:** Jens Klotsche, Dietmar Ferger, Lars Pieper, Jürgen Rehm, Hans-Ulrich Wittchen

**Affiliations:** 1 Institute of Clinical Psychology and Psychotherapy, Technische Universitaet Dresden, Germany; 2 Centre of Clinical Epidemiology and Longitudinal Studies; Faculty of Mathematics and Natural Sciences, Technische Universitaet Dresden, Germany; 3 Institute of Mathematical Stochastic, Technische Universitaet Dresden, Germany; 4 Centre for Addiction and Mental Health, Toronto, Ontario, Canada; 5 Department of Public Health Sciences, University of Toronto, Canada

## Abstract

**Background:**

Epidemiological and clinical studies, often including anthropometric measures, have established obesity as a major risk factor for the development of type 2 diabetes. Appropriate cut-off values for anthropometric parameters are necessary for prediction or decision purposes. The cut-off corresponding to the Youden-Index is often applied in epidemiology and biomedical literature for dichotomizing a continuous risk indicator.

**Methods:**

Using data from a representative large multistage longitudinal epidemiological study in a primary care setting in Germany, this paper explores a novel approach for estimating optimal cut-offs of anthropomorphic parameters for predicting type 2 diabetes based on a discontinuity of a regression function in a nonparametric regression framework.

**Results:**

The resulting cut-off corresponded to values obtained by the Youden Index (maximum of the sum of sensitivity and specificity, minus one), often considered the optimal cut-off in epidemiological and biomedical research. The nonparametric regression based estimator was compared to results obtained by the established methods of the Receiver Operating Characteristic plot in various simulation scenarios and based on bias and root mean square error, yielded excellent finite sample properties.

**Conclusion:**

It is thus recommended that this nonparametric regression approach be considered as valuable alternative when a continuous indicator has to be dichotomized at the Youden Index for prediction or decision purposes.

## Background

Anthropometric parameters such as body mass index (BMI), waist circumference (WC) or waist to height ratio (WHtR) are often applied as indicators of obesity in epidemiological and clinical studies due to their simple application and high correlation with more complex measures [1-3]. As a case in point, decisions made from these parameters are necessarily dependent on statistically reliable derived cut-offs. For example, obesity is an established risk factor for the development of clinical type 2 diabetes [1-4] and also plays a central role in metabolic syndrome according to the National Cholesterol Education Program (NCEP) [5] and the International Diabetes Federation (IDF) [6]. The definitions of metabolic syndrome are based on the same set of risk factors (abdominal obesity, hypertriglyceridemia, low HDL, hypertension, elevated fasting glucose) in the NCEP and by the IDF, but differ in terms of what constitutes the best cut-offs for WC and how to weigh risk factors. Of relevance to our paper, the cut-offs for WC for the IDF classification (≥94 for male and ≥88 for female) of metabolic syndrome are lower than those of the NCEP (>102 for male and >88 for female) and have been a point of contention in recent years [7-9]. This has resulted in the unanswered question of how to best determine cut-offs in situations where the indicator or risk factor is a continuous variable (in this case WC) and the endpoint is dichotomous (in this article type 2 diabetes).

The determination of such cut-offs is often based on the Receiver Operating Characteristic (ROC) plot [10,11] in epidemiological and applied biomedical literature. The cut-off corresponding with the maximum of the sum of sensitivity (= probability of truly diseased people being diagnosed as such) and specificity (= probability of truly non-diseased people being diagnosed as such) is considered optimally related to correct and incorrect classification rates [12]. Furthermore, the cut-off represents the maximum risk difference between the probability of disease outcome (in our case type 2 diabetes) for levels of exposure (here: the anthropometric parameter) below and above the cut-off [13].

In this paper we present a novel statistical approach for estimating the cut-offs for anthropometric parameters in diabetes epidemiology, based on a representative large multistage longitudinal epidemiological study. The resulting estimator yields excellent finite sample properties, when compared with established approaches from the ROC plot. This suggests that when a continuous indicator has to be dichotomized for prediction or decision purposes, this should be the method used in medical research. We will exemplify our method with using different anthropometric measures to predict incidence and prevalence of type 2 diabetes.

## Methods

### Study population

The estimation of cut-offs for selected anthropometric parameters in relation to their association to risk of type 2 diabetes was performed with use of data from the DETECT study. DETECT (Diabetes Cardiovascular Risk-Evaluation: Targets and Essential Data for Commitment of Treatment) is a large nationally representative epidemiologic cross-sectional and prospective longitudinal study in German primary care settings [14,15]. On a target day in September 2003, a nationwide sample of primary care physicians documented, with help of an extensive patient and physician questionnaire, the health state of over 55,000 patients. In addition, a sub-sample of 7,519 patients passed a more intensive standardized laboratory assessment and was followed up after one year in 2004, and after 5 years in 2007/8. All analyses are based on data from this sub-sample. The study was designed to examine the prevalence and comorbidity of diabetes mellitus, hypertension, dyslipidemia, coronary heart disease and associated medical conditions, as well as to determine the frequency of behavioural and clinical risk factors like abdominal obesity, smoking or physical activity and their influence on onset and progression of the considered disease. The study was approved by ethical commission of TU Dresden (AZ: EK149092003; date: 16.9.2003) and all patients gave informed consent.

The cross-sectional analysis of the DETECT sub sample included 6,965 patients (4,120 females, 2,845 males). 554 patients were excluded from the analysis as no data was available for waist circumference, hip circumference, body height or weight.

We included 6,355 patients in the assessment of the longitudinal association between several anthropometric parameters and type 2 diabetes (females/males), with both a follow-up assessment in 2004 or in 2007/8, and information about anthropometric measures at baseline.

### Measures

#### Anthropometric parameter

Waist and hip circumference, body height and weight were measured on the target day by the treating physician, according to standardized written instructions and documented in the physician questionnaire. Waist circumference was measured in centimetres, with a tape between the lowest rib and the iliac crest, while hip circumference was measured in centimetres at the widest circumference around the pelvis. Body mass index was calculated as weight (in kg) divided by squared height (in m^2^). Waist-to-hip ratio was defined as waist divided by hip circumference and waist-to-height ratio, as waist circumference divided by body height.

#### Type 2 diabetes mellitus

Diabetes mellitus was defined exclusively by a doctors' clinical diagnosis being rated as 'definite' in the standardized assessment or the prescription of antidiabetic medication [16]. The classification into type I and type II diabetes also resulted from this diagnosis. For the cut-off estimation, only type II diabetes was used as a disease outcome.

### Statistical approaches for estimating the cut-off obtained at the Youden Index *J*

The Receiver Operating Characteristic (ROC) plot is a widely used approach for determining the optimal cut-off for a continuous risk indicator *X *and a dichotomous disease outcome *Y*, in which the sensitivity is plotted against the 1-specificity. All possible 2 × 2 cross tables can be located on the curve emerging from the dichotomization of the continuous risk indicator *X *for all possible levels *c *of *X *considered as cut-offs. The diagonal line through the points (0,0) and (1,1) displays the random association between *X *and *Y *and is called random ROC. The maximum vertical distance between the ROC curve and random ROC is called the Youden Index [17], *J *= max_*c*_{*sensitivity(c) *+ *specificity(c) *- 1}, for all possible cut-off values *c*. The cut-off *θ *associated with the Youden Index *J *represents the point with the maximum risk difference between the dichotomized continuous risk indicator *X *and dichotomous *Y *[12]. The cut-off *θ *is often referred to as an optimal cut-off for dichotomizing the continuous risk indicator *X *in epidemiology [12,18,19], applied biomedical literature [20,21], and psychology [22,23], when related to total correct and incorrect classification rates [12].

### A novel nonparametric approach for estimating a cut-off

The association between the disease outcome *Y *and the exposure *X *can be modelled in a regression framework where the conditional distribution function *P *[*Y *= *y*|*X *= *x*] is studied for estimating a cut-off *θ*. In contrast, the established estimators for *θ *based on the ROC plot relate the conditional distribution function *P *[*X *≤ *c*|*Y *= 1] (sensitivity) to *P *[*X *> *c*|*Y *= 0] (specificity). The main ideas for the regression related estimator for *θ *are presented here in short; for the exhaustive mathematical theory see Dempfle [24] and Ferger [25].

The following statistical model was studied: (1) the continuous risk indicator *X *with an unknown distribution function, (2) the conditional distribution function *P *[*Y *= *y*|*X *= *x*], *y *∈ {*0, 1*} is Bernoulli distributed with parameter *m*(*x*) and (3) *m*(*x*) = *a1*_{*x *≤ *θ*}_(*X*) + *b1*_{*x *> *θ*}_(*X*), where 1_{A}_(X) denotes the indicator function equals one for x ∈ A and zero otherwise. This nonparametric regression model *Y *= *m*(*x*) + ε = *E *[*Y*|*X *= *x*] with E [ε |*X*] = 0 is well defined by the above quantities. The regression function *m *has a discontinuity or cut-off in *θ *for *a*≠*b*, respectively. The cut-off *θ *indicates the point of change in the probability for disease *Y*. More precisely, the probability for disease is *a *for risk indicator levels *x *≤ *θ *(*a *= *P *[*Y *= 1| *X *≤ θ]) and *b *for risk indicator levels *x *> *θ *(*b *= *P *[*Y *= 1| *X *> θ]).

The Dempfle and Stute (DS) estimator *θ*_*n *_of *θ *is calculated from *n *independent observations (*X*_*i*_, *Y*_*i*_), *i *= 1, ..., *n *of (*X, Y*). For simplicity of notation, the sample is assumed to be ordered by increasing risk indicator levels *X *(*X*_1 _≤ *X*_2 _≤ ... ≤ *X*_*n*_) with the associated Y values denoted by concomitants. The maximum likelihood estimator *θ*_*n *_for the unknown cut-off *θ *is given by(1)

The heuristic idea behind *r*_*n*_(*t*) is comparing the mean of *Y *for levels of *X *smaller than *t *with the mean of *Y *for levels of *X *greater then *t*. The estimator *θ*_*n *_converges to *θ *in probability for *n *→ ∞ with an optimal convergence rate of *n*^-1 ^under weak assumptions on the model, as shown by Dempfle [24] and Ferger [25].

In summary, the algorithm is as follows for calculating the estimator *θ*_*n*_. The functional *r*_*n *_has to be calculated for all *X*_1 _≤ *X*_2 _≤ ... ≤ *X*_*n *_and the estimator *θ*_*n *_is determined as the datum *X*_*c *_associated with the maximum of *r*_*n*_.

### Established methods for estimating the cut-off obtained at Youden Index *J*

The cumulative distribution of *X *for diseased patients (*Y *= 1) is given by *G*: = *P *[*X *≤ *c*|*Y *= 1] and the cumulative distribution of *X *for healthy patients (*Y *= 0) is given by *E*: = *P *[*X *≤ *c*|*Y *= 0], respectively. Therefore, the sensitivity of a diagnostic test is 1 - *G*(*c*) and the specificity is *E*(*c*) for any given cut-off *c*. The Youden Index *J *can be written as the Kolmogorov-Smirnov statistics, *J *= max_*c *_|*E*(*c*) - *G*(*c*)|.

Several methods for estimating the cut-off θ = argmax_*c*_{*sensitivity(c) *+ *specificity(c) *- 1} have been discussed in recent years. These methods tend to assume either normality of the continuous risk indicator, the existence of a transformation function for non-normal distributed risk indicator into a normal distribution, or the estimated cut-off depends on a smoothing parameter by nonparametric methods like kernel smoothing. The work of Fluss and colleagues [26] is a comprehensive overview, which compares several estimation procedures for *J *and *θ *in detail. The ideas and methods of the estimation procedures are presented in short in this paper, technical details can be found elsewhere, for example Fluss et al. [26] or Zou and Hall [27].

#### Normal method (N)

The cut-off *θ *can directly be estimated by

if the assumptions  for the healthy population  for the diseased population (*Y *= 1) hold.

#### Transformed Normal (TN) method

The normality of the distribution of the risk indicator *X *is often questionable in practical applications [26,27]. Zou and Hall [27] have proposed fitting a power transformation of the Box-Cox type [28]. It is assumed that there exists a monotonic transformation function t(λ,.)such that t(λ, *X*) is normally distributed. The parameter *λ *can be determined by maximum likelihood estimation. The cut-off *θ *is estimated by applying the Normal method in the transformed sample following the application of the inverse transformation function.

#### Empirical conditional distribution function (EMP) method

Substituting the conditional distribution functions *G *and *E *by their empirical distribution functions  and  and identifying J is an intuitive method for estimating *θ*.

#### Kernel method (KM)

The Kernel density estimation method can also estimate the conditional distribution functions of *G *and *E*. The functions  and  are determined using the Gaussian kernel function and the bandwidth recommended by Silverman [29] and applied in Fluss et al. [26] according to Zou et al. [30].

## Results

### The equivalence of the cut-off estimated by the DS estimator and obtained at the Youden Index

The cut-off *θ *corresponding to the maximum of the Kolmogorov-Smirnov statistics is given by θ = argmax_*c *_|*E*(*c*) - *G*(*c*)|.

Appel [31] demonstrated in Theorem 1 that a regression function *m *uniformly maximizes the Kolmogorov-Smirnov distance of sensitivity and specificity. The estimation procedure of Dempfle and Stute [24] yields asymptotically the cut-off associated with the maximum of the Kolmogoroff-Smirnov statistic (Youden Index). We refer to the appendix [additional file 1] for a more detailed view to the relation of both estimation strategies.

### Confidence Intervals for the DS estimator

Confidence intervals are important for statistical inference and for the quantification of the precision of point estimates. The limit variable for the maximum likelihood estimator θ_n _is a maximizing point of a compound Poisson process [25]. An analytical solution for the limit distribution of θ_n _for directly obtaining the quantiles of the distribution does not exist. Ferger [25] proposes a Monte-Carlo approximation of the distribution of the smallest and largest maximum point of a compound Poisson process in order to determine its quantiles c_1 _and c_2 _satisfying , where α ∈ (*0, 1*). We refer to Ferger [25] for a detailed illustration of the procedure for estimating confidence intervals for θ_n_.

### Adjusting the cut-off for covariates

The cut-off for the continuous risk indicator *X *can be strongly influenced by covariates. The novel method for estimating a cut-off is based on a nonparametric regression framework. The introduced estimation procedure works only in the bivariate case and it is not possible to directly add an additional covariate to the regression model *Y *= *m*(*x*) + ε = *E *[*Y *| *X *= *x*]. The estimation procedure is based on the comparison of the mean level of the probability of disease for risk indicator levels smaller or equal and greater than a possible cut-off. It is thus not possible to apply this principle to more than one covariate.

Each point on the ROC curve can be quantified as a conditional expectation of a Bernoulli distributed random variable [32]. Therefore, the conditional expectation of the binary random number given a set of covariates could be modelled by an appropriate regression model for binary data, for example using a generalized linear model [33]. The DS estimator could then be applied to the predicted probabilities to get a covariate adjusted estimate for the cut-off of the risk indicator *X*.

### Application to diabetes epidemiology

#### Baseline characteristics of the DETECT laboratory sample

In the sample of N = 7,519, the mean age was 57.7 (SD = 14.4) with 60.1% female. There was a prevalence of 0.5% for type 1 diabetes, 14.2% for type 2 diabetes, 35.5% for hypertension 35.5%, 29.5% for dsylipidemia, and 12.1% for coronary artery disease (table 1). Male patients (18.1%) were more often affected by type 2 diabetes than females (11.5%) [16].

**Table 1:** Characteristics of the DETECT laboratory sample (N = 7,519)

	**Total sample** **(N = 7,519)**		**Male sample** **(N = 3,081)**		**Female sample** **(N = 4,438)**		**patients with type 2 diabetes** **(N = 1,308)**	
	**Mean (SD) or no.**	%*	**Mean (SD) or no.**	%*	**Mean (SD) or no.**	%*	**Mean (SD) or no.**	%*
				
								
*female*	4,438	60.1	-	-	-	-	645	48.9
								
*age*	57.7 (14.4)		58.7 (13.5)		57.0 (14.9)		66.6 (10.0)	
18-44	1,601	24.1	538	19.6	1,063	27.1	42	3.4
45-65	3,378	44.8	1,468	47.4	1,910	43.1	496	37.8
65+	2,540	31.1	1,075	33.0	1,465	29.8	770	58.8
								
*WC*	95.0 (14.9)		101.8 (12.6)		90.3 (14.5)		103.7 (13.5)	
*HC*	105.1 (12.7)		105.9 (10.9)		104.4 (13.8)		110.9 (12.3)	
*BMI*	27.2 (4.9)		27.7 (4.2)		26.7 (5.3)		29.7 (5.0)	
*WHR*	0.91 (0.11)		0.96 (0.08)		0.87 (0.11)		0.94 (0.10)	
*WHtR*	0.56 (0.09)		0.58 (0.07)		0.55 (0.09)		0.62 (0.08)	
								
*type 2 diabetes *†	1,308	14.2	663	18.1	645	11.5	-	-
*hypertension †*	3,078	35.5	1,379	39.5	1,699	32.8	978	71.4
*dyslipidemia †*	2,629	29.5	1,201	33.9	1,428	26.6	720	54.3
*coronary artery disease †*	1,039	12.1	620	18.1	419	8.1	384	29.4
								
*smoking status *‡								
never	3,653	52.9	1,116	39.5	2,537	62.0	624	54.4
current	1,364	20.8	578	21.2	786	20.5	140	12.4
former	1,856	26.3	1,159	39.3	697	17.5	380	33.3
								
*physical activity *‡,¶	4,708	68.0	2,071	72.0	2,637	65.3	738	65.1

BMI = body mass index; HC = Hip circumference; WC = waist circumference; WHR = waist to hip ratio; WHtR = waist to height ratio

Percentages are weighted to age and gender distribution of the DETECT main study (N = 55,518)

† based on physicians diagnosis

‡ based on patients questionnaire (available on http://www.detect-studie.de)

¶physical activity = two or more hours per week

#### Cross-sectional analysis

Table 2 shows the results of cut-off estimations with the use of the DS estimator for several anthropometric parameters for the probability of type 2 diabetes. These cut-offs, based on data from the DETECT study, are useful in discriminating primary care patients with and without type 2 diabetes.

**Table 2:** Estimated cut-off points for anthropometric parameters for predicting type 2 diabetes in cross-sectional analysis by the DS estimator and their longitudinal associations

		cut-off †	95% CI¶	J	PPV ‡	NPV ‡	AUC ‡
***Male***							
	WC	102	101.3 - 103.1	0.26	13.6	94.5	0.67
	HC	106	104.7 - 107.2	0.23	13.7	94.8	0.67
	BMI	27.0	26.6 - 27.8	0.23	12.6	95.1	0.68
	WHR	1.00	0.98 - 1.03	0.16	12.1	92.4	0.56
	WHtR	0.58	0.57 - 0.59	0.30	13.3	94.6	0.69
							
***Female***							
	WC	92	91.4 - 92.8	0.34	10.3	97.0	0.73
	HC	105	104.1 - 105.9	0.29	9.5	97.0	0.70
	BMI	28.0	27.8 - 28.6	0.32	10.8	96.7	0.70
	WHR	0.86	0.85 - 0.88	0.22	8.8	96.7	0.65
	WHtR	0.55	0.54 - 0.57	0.36	10.0	97.9	0.74

AUC = area under curve; BMI = body mass index; CI = confidence interval; HC = Hip circumference; J = Youden Index; NPV = negative predictive value; PPV = positive predictive value; WC = waist circumference; WHR = waist to hip ratio; WHtR = waist to height ratio

† estimated from cross-sectional analyses

‡ PPV, NPV and AUC for the five year incidence of type 2 diabetes using the cut-offs

from cross-sectional analyses

¶The 95% confidence intervals are estimated by a Monte-Carlo approximation of the limit distribution

Except for BMI, the cut-offs of all other examined anthropometric parameters were higher in male patients. The optimal cut-off was 102 cm (male) and 92 cm (female) for WC, with the maximum difference in probability for type 2 diabetes over all age groups. According to the DS estimator the optimal cut-off point for HC was 106 cm for male und 105 cm for female patients. A BMI of 27.0 kg/m^2 ^in male patients and 28.0 kg/m^2 ^in female patients was the best cut-off in relation to the occurrence of type 2 diabetes. Our cut-off estimations for WHR were 1.00 (male) and 0.86 (female) and for WHtR 0.58 (male) and 0.55 (female) respectively.

The cumulative distribution of anthropometric parameter for patients with type 2 diabetes (*G*) and the cumulative distribution of anthropometric parameter for patients without type 2 diabetes (*E*) were investigated by kernel density plots. The distributions are asymptotical normal across all parameter. The DS estimator yields unbiased estimates for normal data as demonstrated in the simulation study (table 3, table 4 and table 5). This fact applies also for smaller values of J (results are not shown).

**Table 3:** Estimated RMSE of estimators for the cut-off *θ *by varying distributional assumptions on the continuous risk indicator *X *for unbalanced sample sizes (60/20), (150/50) and (300,100) and Youden Index *J *= 0.4

		**J = 0.4**					
		**60/20**		**150/50**		**300/100**	
		**Bias**	**RMSE**	**Bias**	**RMSE**	**Bias**	**RMSE**
		
Normal, equal variances		*θ = 6.762*					
	N	0.000	0.096	0.001	0.057	0.003	0.041
	TN	-0.016	0.090	-0.016	0.053	-0.015	0.039
	EMP	0.463	0.513	0.489	0.520	0.499	0.519
	KM	0.019	0.153	0.012	0.106	0.008	0.080
	DS	0.010	0.188	0.007	0.145	0.009	0.125
Normal^ (-1/3)		*θ = 3.068*					
	N	0.370	0.434	0.424	0.453	0.436	0.451
	TN	-0.154	0.242	-0.151	0.199	-0.145	0.171
	EMP	0.697	0.850	0.757	0.852	0.785	0.841
	KM	0.133	0.310	0.083	0.190	0.072	0.151
	DS	-0.007	0.367	-0.004	0.287	0.001	0.238
Mix1		*θ = 11.659*					
	N	-0.260	0.317	-0.254	0.280	-0.250	0.263
	TN	-0.706	0.755	-0.694	0.716	-0.689	0.701
	EMP	-0.357	0.680	-0.226	0.496	-0.132	0.363
	KM	-0.088	0.365	-0.092	0.269	-0.088	0.213
	DS	-0.292	0.593	-0.176	0.450	-0.107	0.346
Mix2		*θ = 10.665*					
	N	0.532	0.575	0.545	0.562	0.551	0.559
	TN	-0.050	0.196	-0.055	0.133	-0.055	0.101
	EMP	-0.058	0.419	-0.026	0.332	-0.007	0.254
	KM	0.150	0.393	0.102	0.265	0.069	0.186
	DS	-0.023	0.388	-0.002	0.318	0.007	0.246
Mix3		*θ = 14.244*					
	N	-1.831	1.854	-1.840	1.850	-1.850	1.854
	TN	-1.209	1.246	-1.213	1.228	-1.211	1.219
	EMP	-0.947	1.539	-1.013	1.614	-0.867	1.502
	KM	-1.700	2.059	-1.754	2.098	-1.672	2.043
	DS	-1.059	1.589	-1.084	1.641	-0.919	1.531
Mix4		*θ = 14.430*					
	N	-1.823	1.849	-1.836	1.846	-1.838	1.843
	TN	-1.133	1.175	-1.129	1.145	-1.141	1.149
	EMP	-0.698	1.418	-0.478	1.174	-0.352	0.992
	KM	-1.408	1.876	-1.144	1.636	-0.924	1.450
	DS	-0.799	1.447	-0.549	1.197	-0.391	1.018

DS = Dempfle and Stute estimator; EMP = empirical conditional distribution function method; J = Youden Index;

KM = Kernel method; N = Normal method; TN = transformed normal method

**Table 4:** Estimated RMSE of estimators for the cut-off *θ *by varying distributional assumptions on the continuous risk indicator *X *for unbalanced sample sizes (60/20), (150/50) and (300,100) and Youden Index *J *= 0.6

		**J = 0.6**					
		**60/20**		**150/50**		**300/100**	
		**Bias**	**RMSE**	**Bias**	**RMSE**	**Bias**	**RMSE**
		
Normal, equal variances		*θ = 6.921*					
	N	0.007	0.066	0.003	0.043	0.001	0.030
	TN	-0.035	0.078	-0.036	0.057	-0.037	0.048
	EMP	0.281	0.329	0.301	0.325	0.313	0.329
	KM	0.006	0.107	0.010	0.077	0.007	0.055
	DS	0.007	0.154	0.012	0.125	0.011	0.097
Normal^ (-1/3)		*θ = 3.201*					
	N	0.442	0.498	0.484	0.509	0.514	0.530
	TN	-0.244	0.282	-0.257	0.271	-0.263	0.270
	EMP	0.539	0.688	0.567	0.650	0.624	0.678
	KM	0.193	0.307	0.147	0.212	0.116	0.166
	DS	0.001	0.344	0.005	0.257	0.009	0.216
Mix1		*θ = 11.103*					
	N	0.399	0.440	0.416	0.432	0.415	0.423
	TN	-0.109	0.216	-0.085	0.144	-0.088	0.123
	EMP	-0.093	0.430	-0.049	0.322	-0.036	0.257
	KM	0.301	0.419	0.243	0.308	0.192	0.244
	DS	-0.036	0.406	-0.026	0.307	-0.018	0.250
Mix2		*θ = 10.900*					
	N	0.379	0.430	0.395	0.412	0.400	0.409
	TN	-0.067	0.169	-0.074	0.117	-0.067	0.094
	EMP	-0.047	0.337	-0.031	0.253	-0.016	0.194
	KM	0.083	0.248	0.040	0.153	0.036	0.111
	DS	-0.010	0.312	-0.016	0.239	-0.003	0.184
Mix3		*θ = 13.184*					
	N	0.378	0.459	0.396	0.429	0.393	0.409
	TN	-0.013	0.262	-0.012	0.168	-0.014	0.116
	EMP	-0.089	0.622	-0.048	0.472	-0.042	0.392
	KM	0.211	0.594	0.179	0.408	0.135	0.300
	DS	-0.027	0.574	-0.019	0.444	-0.013	0.373
Mix4		*θ = 12.874*					
	N	0.715	0.766	0.717	0.737	0.717	0.727
	TN	0.296	0.405	0.294	0.341	0.294	0.318
	EMP	0.002	0.631	0.017	0.481	0.011	0.391
	KM	0.306	0.665	0.200	0.448	0.146	0.321
	DS	0.066	0.606	0.061	0.469	0.047	0.380

DS = Dempfle and Stute estimator; EMP = empirical conditional distribution function method; J = Youden Index;

KM = Kernel method; N = Normal method; TN = transformed normal method

**Table 5:** Estimated RMSE of estimators for the cut-off *θ *by varying distributional assumptions on the continuous risk indicator *X *for unbalanced sample sizes (60/20), (150/50) and (300,100) and Youden Index *J *= 0.8

		**J = 0.8**					
		**60/20**		**150/50**		**300/100**	
		**Bias**	**RMSE**	**Bias**	**RMSE**	**Bias**	**RMSE**
		
Normal, equal variances		*θ = 7.141*					
	N	0.009	0.072	0.000	0.045	0.001	0.030
	TN	-0.034	0.093	-0.043	0.069	-0.043	0.057
	EMP	0.159	0.218	0.183	0.218	0.196	0.214
	KM	0.007	0.097	-0.004	0.065	-0.001	0.048
	DS	0.006	0.139	0.005	0.105	0.008	0.086
Normal^ (-1/3)		*θ = 3.525*					
	N	0.406	0.480	0.443	0.478	0.465	0.489
	TN	-0.220	0.326	-0.252	0.289	-0.263	0.280
	EMP	0.425	0.637	0.480	0.595	0.484	0.551
	KM	0.299	0.424	0.197	0.267	0.156	0.200
	DS	0.014	0.375	0.026	0.287	0.011	0.225
Mix1		*θ = 11.373*					
	N	0.298	0.352	0.302	0.325	0.307	0.318
	TN	0.008	0.183	0.014	0.117	0.015	0.082
	EMP	-0.094	0.346	-0.040	0.246	-0.032	0.190
	KM	0.154	0.284	0.108	0.183	0.084	0.135
	DS	-0.063	0.310	-0.013	0.232	-0.011	0.185
Mix2		*θ = 11.305*					
	N	0.150	0.225	0.173	0.203	0.169	0.185
	TN	-0.087	0.164	-0.077	0.116	-0.081	0.104
	EMP	-0.074	0.295	-0.028	0.214	-0.022	0.170
	KM	0.014	0.180	0.017	0.118	0.005	0.088
	DS	-0.052	0.269	-0.008	0.202	-0.003	0.167
Mix3		*θ = 13.837*					
	N	0.124	0.268	0.124	0.196	0.124	0.166
	TN	-0.099	0.263	-0.102	0.185	-0.103	0.152
	EMP	-0.096	0.407	-0.038	0.283	-0.020	0.221
	KM	0.037	0.306	0.023	0.183	0.018	0.126
	DS	-0.066	0.376	-0.009	0.269	-0.002	0.217
Mix4		*θ = 13.887*					
	N	0.159	0.303	0.152	0.223	0.156	0.193
	TN	-0.055	0.267	-0.070	0.177	-0.067	0.135
	EMP	-0.068	0.458	-0.040	0.302	-0.008	0.242
	KM	-0.002	0.335	-0.026	0.208	-0.020	0.143
	DS	-0.030	0.419	-0.021	0.292	-0.003	0.237

DS = Dempfle and Stute estimator; EMP = empirical conditional distribution function method; J = Youden Index;

KM = Kernel method; N = Normal method; TN = transformed normal method

#### Longitudinal analysis

In a second step, we used the estimated cut-offs of the different anthropometric parameter to determine their predictive values for the five year incidence of type 2 diabetes. Wherein we calculated the positive and negative predictive value as well as the area under the receiver operator curve (AUC). We found a five year type 2 diabetes incidence of 6.7%, in which male patients (8.5%) were more frequently affected than females (5.6%).

The negative predictive value for the incidence of type 2 diabetes while applying the cut-offs ranged between 92.4 for WHR and 95.1 for BMI in male patients, and 96.7 and 97.9 in female patients, respectively. The positive predictive value of the cut-offs for the researched parameters of incident type 2 diabetes ranged between 12.1 (WHR) and 13.7 (HC) in male patients and 8.8 (WHR) and 10.8 (BMI) in female patients.

### Simulation study on different procedures to estimate the best cut-off for diabetes

#### Design of the Monte Carlo simulation study

We compared the DS estimator (24) with the estimation procedures presented by Fluss et al. [26]. The design of our simulation study was similar when compared to that of Fluss et al. [26], except that the outcome variable *Y *was also modelled as a realization of a Bernoulli distributed random number, which broadened the design of the simulation study. When simulating the following distributions of *X*: (1) the conditional distribution of *P *[*X *≤ *c*|*Y *= 0] followed a  - distribution, the conditional distribution of *P *[*X *≤ *c*|*Y *= 1] followed a  - distribution (as an example for symmetric distributions); (2) *P *[*X *≤ *c*|*Y *= 0] followed a  - distribution and *P *[*X *≤ *c*|*Y *= 1] followed a  - distribution (as an example for skewed distributions); (3) the case of a bimodal distribution of the conditional distributions *P *[*X *≤ *c*|*Y *= 0] and *P *[*X *≤ *c*|*Y *= 1] was covered by a mixture of two normal distributions .

Detailed information about the parameter settings can be found in Table 6. The estimated cut-off *θ *was chosen to correspond with the Youden Index *J *∈ {0.4, 0.6, 0.8}. The simulation study used balanced and unbalanced sample sizes ((20/20), (50,50), (100/100)) and ((60/20), (150,50), (300/100)) respectively, representing the number of (healthy subjects (*Y *= 0)/diseased subjects (*Y *= 1)).

**Table 6:** Parameter values of the distributions used in the Monte-Carlo study

	p_H_	p_D_	μ_H1_	σ^2^_H1_	μ_H2_	σ^2^_H2_	σ^2^_D1_	σ^2^_D2_	μ_D1_			μ_D2_
									J = 0.4	J = 0.6	J = 0.8	
*Distributions of X*												
Normal, equal variances			6.5	0.25			0.25		7.024	7.342	7.782	
Normal, non equal variances			6.5	0.09			0.25		6.873	7.143	7.505	
Normal^-1/3^			3.5	0.09			0.25		3.127	2.857	2.495	
Mix1	1	0.5	10	1			1	5	9.980	11.120	12.160	μ_D1_+4
Mix2	1	0.8	10	1			1	5	10.850	11.530	12.440	μ_D1_+4
Mix3	0.5	0.5	10	1	13	1	1	5	12.390	13.580	14.740	μ_D1_+4
Mix4	0.5	0.5	10	1	13	1.5	1	5	12.240	13.580	14.910	μ_D1_+4
Mix5	0.5	0.8	10	1	13	1	1	5	12.640	13.900	15.000	μ_D1_+4
Mix6	0.5	0.8	10	1	13	1.5	1	5	12.510	13.860	15.160	μ_D1_+4

J = Youden Index; Mix = mixture of two normal distributions; Normal = normal distribution

The algorithms for estimating the cut-offs and the simulation trials were implemented in STATA 10.1 [34] with a double precision number format. The number of Monte-Carlo replications was set to 5000. The performance of several estimation methods were compared by root mean square error (RMSE); this is a relevant measure for studying the performance of biased estimators, as it combines the systematic bias and the random error of an estimator.

#### Results of the simulation study

The results of the simulation study for unbalanced sample sizes are displayed in table 3 for *J *= 0.4, in table 4 for *J *= 0.6 and in table 5 for *J *= 0.8. The bias is smallest for N, TN and DS in the case of normal distributed marginal distributions with equal variances as well as unequal variances. The DS method produces a larger RMSE compared with N and TN as expected for a nonparametric method. The EMP yields a considerable bias for normal data and skewed data (normal^(-1/3)). The DS produces the smallest bias in the skewed data scenario, while N and TN, KM and EMP perform worse. The results for mix1, mix3, mix5 and mix6 are quite similar. The DS produces small bias for cases *J *≥ 0.6 and performs in most cases best. Again, the RMSE is increased for DS compared to the other well performing methods in the respective simulation scenario. The DS method is biased when *J *= 0.4 which appears with a small risk difference between the probability of disease outcome for levels of exposure below and above the cut-off. The DS estimator results in smallest bias for the mix2 simulation scenario. There is a consistent pattern across all simulation scenarios. The performance of DS is improved with increasing sample sizes in all simulation trials, as expected for a nonparametric estimator. It is remarkable that N, TN, EMP, KM and DS estimates the valley of *P *[*X *≤ *c*|*Y *= 0] instead of the cut-off in Mix3 and Mix4 for *J *= 0.4. A more detailed analysis of the simulation results shows that this phenomenon only occurs in very few cases in the other simulation scenarios. The simulation results for the case of balanced sample sizes are comparable to the data presented in table 3, table 4 and table 5.

To summarize the results of the simulation study, the estimator DS performs well in most simulation trials in case of *J *≥ 0.6. The performance is getting better for larger sample sizes measured by RMSE. It is remarkable that DS also results in acceptable bias for small sample sizes. This suggests that this estimator should provide an alternative method for estimating the cut-off associated with the Youden Index in epidemiological research beside the existing methods.

## Discussion

The present study addresses the estimation of the cut-off associated with the Youden Index. We have presented an approach for obtaining optimal cut-offs and demonstrated that this approach has excellent statistical properties in a Monte-Carlo study. The DS estimator is easy to implement and should be applied for estimating a cut-off in epidemiological research.

**Figure 1:**
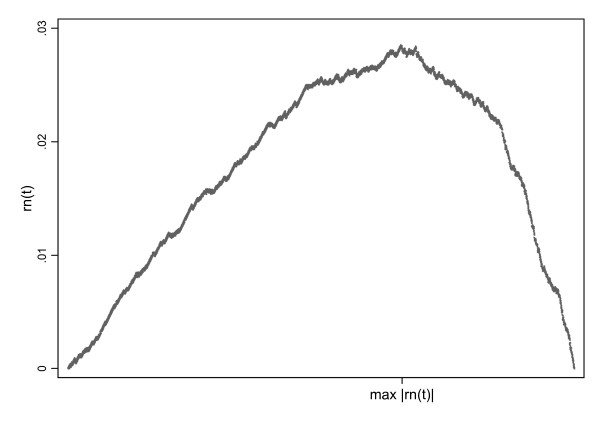
**Triangular structure of the function *r*_*n *_for the association of waist circumference and type 2 diabetes for males**.

This method of DS also has minimal assumptions on the underlying statistical model, as it is nonparametric. It should also be emphasized that the procedure estimates *θ *without any smoothing technique, such as kernel regression [35,36] or local polynomial regression [37]. Therefore, the ability to detect a possible cut-off in the data does not depend on any smoothing window or bandwidth. Essentially the correct estimate of the cut-off only depends on the triangular shape of the function *r*_*n*_. In fact, this function is flattened by decreasing the risk difference for the probability of disease for levels of *X *below and above *θ*. The function *r*_*n *_is a straight line in the case of a risk difference of zero. We thus recommend plotting the function *r*_*n *_in a practical application and assessing its triangular form when using this method (figure 1).

The shape of the estimated regression function *m *was assumed to be a step function with a discontinuity in θ and the magnitude of |*a *- *b*|. This assumption, as mentioned in the presentation of the Dempfle and Stute [24], appears to be very restrictive, In actuality, this special shape of the regression function is the most challenging issue, because a monotonic increasing regression function *m *with *m *≤ *a *for *X *≤ θ and *m *≥ *b *for *X *> θ would boost the difference between the estimated conditional expectations for the risk indicator levels. This estimation procedure only assumes monotonic increase or decrease of risk for disease for increasing or decreasing risk indicator levels, a common assumption. The mathematical theory for a general *m *can be found in Ferger [25].

The aim of this study was to introduce a new approach for estimating optimal cut-offs of several anthropometric parameters for the probability of type 2 diabetes in a German primary care population. However, as people are gaining more weight becoming more obese with age, appropriate cut-offs for anthropometric parameters are important for deciding whether the level of a parameter is associated with an increased risk for type 2 diabetes for the application in everyday life.

The estimated WC value for the female group (92 cm) was above the NCEP ATP III (women > 88 cm) (5), while the WC value for men (102 cm) precisely matched the NCEP cut-off. In both groups, the cut-offs were above the International Diabetes Federation (men > 94 cm; woman >80 cm) (6) criteria for the metabolic syndrome. An explanation for higher cut-off values in our estimations could be that the sample of primary care patients from the DETECT study may have had an increased risk for disease compared with the general population, due to the setting of the study. In this example, cut-offs of different anthropometric parameters represent tools for physicians to make decisions about type 2 diabetes risk. This has implications for further examinations and treatment of concerned patients.

## Conclusion

The Dempfle and Stute [24] estimator provides a flexible nonparametric approach for estimating a cut-off for a continuous risk indicator based on the argument of maximum risk difference between the dichotomized continuous risk indicator *X *and dichotomous outcome *Y*. The estimation procedure avoids artificial parametric assumptions on the data as it is nonparametric. In addition to the results of the simulation study also suggest that the estimator could be considered as an alternative in epidemiology and clinical studies, when a continuous indicator needs to be dichotomized for prediction or decision purposes.

## Competing interests

The authors declare that they have no competing interests.

## Authors' contributions

Details of the manuscript's creation are as follows. JK developed the ideas of the paper and prepared the manuscript. JK also proved the equivalence of the cut-off estimated by the estimator of Dempfle and Stute and the cut-off obtained at the Youden index. LP prepared the materials, methods and results section concerning the data of the DETECT study. DF supervised the theoretical mathematical calculations and prepared parts of it. JR had substantial impact on the application of the Demple and Stute estimator to diabetes epidemiology and critically revised the final version of the manuscript. Finally, HUW designed the DETECT study and directed its implementation. We also ensure that this manuscript has not been published or submitted elsewhere. All authors read and approved the final manuscript.

## Pre-publication history

The pre-publication history for this paper can be accessed here:

http://www.biomedcentral.com/1471-2288/9/63/prepub

## Supplementary Material

Additional file 1 **Appendix**. The equivalence of the cut-off estimated by the DS estimator and obtained at the Youden Index. Click here for file
