# Supplementary material for: oA novel nonparametric approach for estimating cut-offs in continuous risk indicators with application to diabetes epidemiology
Source: BMC Med Res Methodol. 2009 Sep 10;9:63. doi: 10.1186/1471-2288-9-63 (PMC2754490; doi:10.1186/1471-2288-9-63)
Supplement: Additional file 1 — Appendix. The equivalence of the cut-off estimated by the DS estimator and obtained at the Youden Index. [file 1471-2288-9-63-S1.doc]

APPENDIX

*Lemma:* For the following model

(1) $\left(X_{i},Y_{i}\right)\sim\left(X,Y\right)$ are independent and identical distributed random vectors (*1≤i≤n)*

(2) *X* has an unknown marginal distribution function denoted by *F*

(3) the conditional distribution function $P\left[Y\in\left\{0,1\right\}\text{ }\left|X=x\right.\right]$ is Bernoulli distributed with parameter *m(x)*

(4) $m(x)=a1_{\left\{x\leq\theta\right\}}(x)+b1_{\left\{x>\theta\right\}}(x)$

The Dempfle and Stute estimator and the cut-off obtained at the Youden Index are asymptotically equivalent.

*Sketch of the Proof:*

Let us introduce $H_{n}\left(c\right):=\frac{1}{n}\sum_{i=1}^{n} 1_{\left\{X_{i}\leq c\right\}}Y_{i}$, $F_{n}\left(c\right):=\frac{1}{n}\sum_{i=1}^{n} 1_{\left\{X_{i}\leq c\right\}}$ and $M_{n}:=\frac{1}{n}\sum_{i=1}^{n} Y_{i}$ for any real number *c*. The false negative rate is given by $\frac{\sum_{i=1}^{n} 1_{\left\{X_{i}\leq c\right\}}Y_{i}}{\sum_{i=1}^{n} Y_{i}}$ and the true negative rate by $\frac{\sum_{i=1}^{n} 1_{\left\{X_{i}\leq c\right\}}\left(1-Y_{i}\right)}{\sum_{i=1}^{n} \left(1-Y_{i}\right)}$ for empirical data $\left(X_{i},Y_{i}\right)$, (*1≤i≤n)* (for example, we confer to Appel [31]).

We have

The expectation of *H_n_(c)* is equal to $H=\int_{-\infty}^{c} m\left(x\right)F\left(dx\right)$ (Ferger [38]), the empirical distribution function *F_n_(c)* converges almost sure to *F(c)* by the Glivenko-Cantelli Theorem and the expectation of the mean value *M_n_* is *E[Y]*. Furthermore, *M_n_* does not depend on the value *c*, therefore this quantity can be omitted from our considerations of the argmax-functional. Applying the Theorem of Cramer and Slutzky and the Argmax continuous mapping theorem of Ferger [38] we have for$n\to\infty$

| $\mathrm{argmax}_{c}\left\vert\left. H_{n}\left(c\right)-F_{n}\left(c\right)M_{n} \right\vert\right. \to\mathrm{argmax}_{c}\left\vert H\left(c\right)-F\left(c\right)E\left[Y\right]\right\vert$ | (1) |
| --- | --- |

.

On the other side we have

$$\begin{matrix} \mathrm{argmax}_{c} \left|\left. E\left[r_{n}\left(c\right)\right] \right|\right. \\ =\mathrm{argmax}_{c} \left|\left. E\left[\frac{1}{n-1} \sum_{i=1}^{n} 1_{\left\{X_{i}\leq c\right\}}\left(Y_{i}-\overline{Y}_{n}\right)\right] \right|\right. \\ = \mathrm{argmax}_{c} \left|\left. \frac{n}{n-1} \left(E\left[\frac{1}{n}\sum_{i=1}^{n} 1_{\left\{X_{i}\leq c\right\}}Y_{i}\right] - E\left[\frac{1}{n}\sum_{i=1}^{n} 1_{\left\{X_{i}\leq c\right\}}\frac{1}{n}\sum_{i=1}^{n} Y_{i}\right] \right) \right|\right. \\ = \mathrm{argmax}_{c} \left|\left. \frac{n}{n-1}\left( E\left[\frac{1}{n}\sum_{i=1}^{n} 1_{\left\{X_{i}\leq c\right\}}Y_{i}\right] - E\left[\frac{1}{n^{2}}\sum_{i=1}^{n} \sum_{j=1,i\neq j}^{n} 1_{\left\{X_{i}\leq c\right\}}Y_{j}\right] - E\left[\frac{1}{n^{2}}\sum_{i=1}^{n} 1_{\left\{X_{i}\leq c\right\}}Y_{i}\right]\right) \right|\right. \end{matrix}$$

The observations $1_{\left\{X_{i}\leq c\right\}}$ and $Y_{j}$are independent for $i\neq j$, thus

$$\begin{matrix} = \mathrm{argmax}_{c} \left|\left. \frac{n}{n-1}\left( H\left(c\right) - \frac{n\left(n-1\right)}{n^{2}} F\left(c\right) E\left[Y\right] - \frac{1}{n}H\left(c\right)\right) \right|\right. \\ \\ = \mathrm{argmax}_{c} \left|\left. \frac{n}{n-1}\left( H\left(c\right) - \left(1-\frac{1}{n}\right) F\left(c\right) E\left[Y\right]- \frac{1}{n}H\left(c\right)\right) \right|\right. \\ \\ = \mathrm{argmax}_{c} \left|\left. \frac{n}{n-1}\left( H\left(c\right) - F\left(c\right) E\left[Y\right] - \frac{1}{n}\left(H\left(c\right)-F\left(c\right) E\left[Y\right]\right)\right) \right|\right. \end{matrix}$$

It follows for $n\to\infty$

| $\begin{matrix} \mathrm{argmax}_{c} \left\vert\left. \frac{n}{n-1}\left( H\left(c\right) - F\left(c\right) E\left[Y\right] - \frac{1}{n}\left(H\left(c\right)-F\left(c\right) E\left[Y\right]\right)\right) \right\vert\right. \\ \to\mathrm{argmax}_{c} \left\vert\left. H\left(c\right)-F\left(c\right) E\left[Y\right] \right\vert\right. \end{matrix}$ | (2) |
| --- | --- |

This shows the asymptotic equality of the Dempfle and Stute estimator and cut-off obtained at the Youden Index.
